# Editorial

**Published:** 1874-11

**Authors:** 


### Editorial.

In the October Number of the JOURNAL we published, under the head of Correspondence, a communication signed "Regular," anent the matter of Ethics, which appears to have called out an answering echo in the mind of at least one of our readers, whose article over the signature of a "Country Physician—Regular" is published herein.

While we shall continue with much pleasure to publish to the world the opinions of our readers so long as they are within the bounds of reason, we must disclaim all responsibility for the same, directly or indirectly. The JOURNAL is simply the medium of communication for its correspondents, but endorses neither their ethics, logic, nor grammar. H.

***Sanitary.***

The Mortality Report for the month of September, published herein, exhibits a very gratifying aspect of the sanitary condition of the city. The report shows a decrease of 411 deaths in comparison with the mortality of the preceding month; and what is still more satisfactory, a decrease of 199 when compared with the list for the corresponding month of last year; this decrease occurring coincidently with an increase in the mean temperature of 4.3°, and likewise an increase in the population. This decreased mortality has been apparent in people of all ages, but principally in children under five years of age. The approaching cold weather will have the effect to still farther decrease the mortality.

---

